# Machine learning-based assessment of seizure risk predictors in myelomeningocele patients: A single-center retrospective cohort study

**DOI:** 10.5339/qmj.2025.15

**Published:** 2025-03-13

**Authors:** Maher Al Rifai, Sultan Jarrar, Mohammad Barbarawi, Mohammad Jamous, Suleiman Daoud, Amer Jaradat, Owais Ghammaz, Bashar Hatem Abulsebaa, Qutaiba Alsumadi, Tala Ali Shibli, Ahmad Osamah Alqudah

**Affiliations:** ^1^Jordan University of Science and Technology, Ar-Ramtha, Irbid, Jordan *Correspondence: Maher Al Rifai. Email: Alrifaimd@gmail.com

**Keywords:** Myelomeningocele, seizure, machine learning, ventriculoperitoneal shunt

## Abstract

**Background:**

Myelomeningocele (MMC) is a severe congenital malformation of the CNS (central nervous system) that often leads to seizures due to factors such as shunt complications and hydrocephalus. This study aims to develop a machine learning model to predict the likelihood of seizures in MMC patients by analyzing various predictors.

**Methods:**

This retrospective study involved 103 MMC patients. Factors such as demographics, MMC location, shunt history, and imaging were analyzed using the random forest classifier, the support vector classifier, and logistic regression. Model performance was assessed through bootstrap estimates, cross-validation, classification reports, and area under the curve (AUC).

**Results:**

Of the evaluated patients, 11 experienced seizures. The key influencing factors included gestational age, sacral location, hydrocephalus, shunt history, and corpus callosum dysgenesis. Machine learning (ML) models predicted seizure risk with an accuracy of 86–92% and an AUC ranging from 0.764 to 0.865. Significant predictors were imaging findings, shunt infection history, and gestational age.

**Conclusion:**

ML models effectively predict seizure risk in MMC patients, with certain variables showing strong associations and significant impact.

## Introduction

Myelomeningocele (MMC), also known as open spina bifida, is a serious congenital malformation of the central nervous system, posing significant challenges in terms of morbidity and mortality. This prevalent birth defect has a profound and lasting impact, leading to significant lifelong morbidity.^1^ On a global scale, approximately 300,000 infants are born annually with neural tube defects. The incidence of such defects varies worldwide, ranging from 0.17 to 6.39 per 1,000 live births. In Jordan, the estimated incidence of congenital malformations is 1.1 per 1,000 live births, highlighting the regional impact on the Jordanian population.^2^

The majority of individuals diagnosed with MMC also experience hydrocephalus (HC), which is characterized by the abnormal and progressive accumulation of cerebrospinal fluid in the brain's ventricles. This condition, along with associated factors such as Chiari II malformation, symptoms of tethered cord, lower extremity contractures, and other related brain malformations, significantly influences the overall severity of patient outcomes. The introduction of the ventriculoperitoneal (VP) shunt has enhanced the prospect of long-term survival for infants with open spina bifida. However, shunted HC is prevalent and correlates with cognitive impairments, particularly impacting executive functions.^3–7^ The VP shunt has proven effective in prolonging the lifespan of individuals with HC. However, it requires meticulous maintenance to prevent complications associated with the shunt.^7^ Annually, approximately 14,000 shunt revisions are performed, reflecting the elevated incidence of shunt complications. These complications not only contribute to patient morbidity but also impose a considerable financial burden on healthcare systems. Moreover, similar to any procedure involving the implantation of foreign bodies, there are inherent risks of infection and mechanical dysfunction that can result in serious repercussions.^8,9^

Seizures are commonly observed in children born with MMC. The increased incidence of seizures in individuals with MMC and HC may be attributed to various factors, including minor cortical injury during shunt insertion, complications such as infections or shunt malfunctions, and the underlying pathology associated with the development of HC.^10^ MMC is associated with the occurrence of seizures, particularly in children, where the development of seizures is often associated with the presence of other cerebral malformations and mental retardation. Moreover, the risk of seizures is increased by factors such as shunt dysfunction and infection.^11^ Several studies have previously identified the predictors of seizures in MMC patients. Some findings have indicated a reduced incidence of seizures in MMC patients who do not require a VP shunt, in stark contrast to shunted patients who exhibit a significantly higher incidence of seizures. Other predictors identified in the literature that may influence seizure risk include falx dysgenesis, lumbar MMC, cortical atrophy, and mental retardation.^10,12,13^

To the best of our knowledge, there has been no prior research using a machine learning model to predict the risk of seizures in patients with MMC. Although previous studies have explored the descriptive characteristics of seizures and identified potential predictors in this patient population, none have used a machine learning approach for a comprehensive risk assessment and prediction. This study marks the pioneering effort in developing a robust machine learning model for predicting seizures in patients with a history of MMC. To the best of our knowledge, no earlier research has pursued this avenue, making this study the first of its kind to explore the potential of machine learning in predicting seizures among the MMC patient population.

## Methods

### Data collection and patient characteristics

We conducted a retrospective, observational, single-center cohort study at King Abdullah University Hospital (KAUH), the main tertiary care hospital in Northern Jordan. The study adhered to the guidelines set forth by the Transparent Reporting of a Multivariable Prediction Model for Individual Prognosis or Diagnosis (TRIPOD) statement, which provides guidelines for reporting and developing predictive models.^14^ The Research Committee of the Faculty of Medicine and the Institutional Review Board at Jordan University of Science and Technology approved the study, and the Institutional Review Board provided the ethical approval (2023/164/88). The ethics committee approved a waiver of consent from the patients as the study did not involve any therapeutic interventions and the outcomes planned were routinely registered for all patients. The study included all patients diagnosed with MMC at KAUH between June 2009 and September 2023. We conducted a comprehensive review of patient charts to collect information on several key factors: demographic information (age and gender), gestational age (full-term defined as children born ≥ 37 weeks or preterm defined as children born < 37 weeks), mode of delivery (normal vaginal delivery or cesarean section), level of MMC defect (thoracic, thoracolumbar, lumbar, lumbosacral, or sacral), presence of HC, shunt history (shunt placement, number of shunt revisions, or history of shunt infection), shunt location (frontal, parietal, or occipital), and presence of seizures. For patients with a history of seizures, we collected further data, including seizure type (generalized or focal seizures), the total number of seizures, the age of onset of the first seizure, and current medications taken by the patient. Furthermore, we reviewed neuroimaging data, including findings from computed tomography and magnetic resonance imaging, to identify any central nervous system abnormalities.

### Machine learning analysis

**Figure 1.**
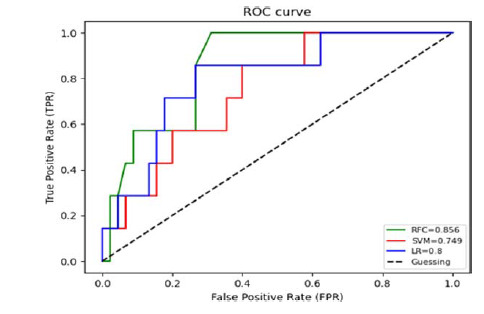
ROC curve for ML algorithms.

Three machine learning (ML) ensembles were used in this study: the random forest classifier (RFC), the support vector classifier (SVC), and logistic regression (LR). These algorithms were selected due to their effectiveness in handling complex, multidimensional datasets and their ability to model nonlinear relationships. The RFC is a decision tree-based ML model, where each node of the decision tree divides the dataset into two groups using a cutoff value within one of the features. By creating an ensemble of randomized decision trees, each of which overfits the data and aggregates the results to achieve improved classification, the RFC technique mitigates the impact of the overfitting problem. The SVC is a powerful supervised ML technique that aims to find the optimal hyperplane to separate data into different classes. It is well suited for both classification and regression applications. In this approach, trees for prediction are sequentially constructed, with each subsequent tree designed to reduce errors from its predecessors. Subsequently, ML models were trained on a dataset with a binary classification output that predicted the target variable Presence of Seizures using 27 features including demographic information, perinatal history, the level of MMC, the presence of HC, shunt history, shunt location, and neuroimaging findings. The dataset was then randomly divided into a training set (*n* = 72) and a testing set (*n* = 31) in a 7:3 ratio. In this study, missing values were estimated by applying the imputation method, which involved filling all null variables with zero values using the “fillna()” method with the “inplace = True” parameter. The contribution of each feature in predicting SFS status was calculated using the permutation importance method, in which a greater decrease in mean accuracy represents higher importance in the model's predictions. Receiver-operating characteristic (ROC) curves and area under the curve (AUC) scores were calculated to evaluate the discriminatory power of different models. The roc_curve function from the sklearn.metrics module was used to compute the FPR (false positive rate) and TPR (true positive rate) for each model. Predicted probabilities for the positive class were obtained using the predict_proba method of each model. AUC scores were calculated using the roc_auc_score function. A custom plotting function plot_roc_curve was defined to visualize the ROC curves for multiple models. Additionally, the model's performance was evaluated using a mean bootstrap estimate with a 95% confidence interval, 10-fold cross-validation, and a classification report for precision, recall, and fig1-score. All ML implementations were processed using the scikit-learn 0.18 package in Python.

### Statistical analysis

Data analyses were performed using the IBM Statistical Package for the Social Sciences (SPSS) software for Windows, version 26.0. Descriptive measures were reported as means ± standard deviations for continuous data that met the normality assumption, according to the Shapiro–Wilk test. In cases where the normality assumption was not satisfied, medians with the first and third quartiles (Q1–Q3) were used. Categorical data were reported as frequencies and percentages (%). Continuous data were compared using the Student's t test for normally distributed variables, while the Mann–Whitney U-test was used for those that were not normally distributed. For categorical data, comparisons were made using the χ^2^ test or Fisher's exact test if any cell had an expected count of less than 5. Effect sizes for numerical variables were calculated using Cohen's d for normally distributed numerical variables, while rank–biserial correlation was used if the normality assumption was violated. Effect sizes for categorical variables were calculated using Cramér's V. The selection of variables included in the model was based on a separate bivariate analysis, which included all variables that yielded a *p* value of < 0.1. The goodness of fit was assessed using Nagelkerke R2. The variables in the model were evaluated for multicollinearity using the variance inflation factor. Statistical significance was considered at a two-sided *p* value of ≤ 0.05. Effect sizes were considered statistically significant4 at *p* values ≥ 0.3.

## Results

Our analysis of the demographic and clinical characteristics of patients with MMC, as summarized in Table 1, revealed several significant findings. Notably, there was a tendency for a higher proportion of females among patients with seizures (81.8%) compared to those without seizures (56.5%), although this difference was not statistically significant (*p* = 0.107). The median age of patients with seizures was 8 years, which was similar to that of patients without seizures (7 years), with no statistically significant difference (*p* = 0.945). However, patients with seizures had a significantly higher prevalence of preterm birth (60%) compared to those without seizures (20.5%) (*p* = 0.018). HC patients with seizures had a significantly higher rate of HC (100%) compared to those without seizures (70.7%) (*p* = 0.036). Patients with seizures had a significantly higher rate of shunt placement (100%) compared to those without seizures (70.7%), with a *p* value of 0.036. Additionally, patients with seizures had a higher median number of shunt revisions (2) compared to those without seizures (0) (*p* ≤ 0.001), with a significant effect size of 0.573. Furthermore, a history of shunt infection was significantly more common among patients with seizures (72.7%) than in those without seizures (26.1%) (*p* = 0.002), with a significant effect size of 0.311. Patients with seizures did not differ significantly from those without seizures in terms of the level of MMC, with *p* values above 0.05. However, there was a significantly higher prevalence of seizures (27.4%) in patients with sacral MMC compared to those without seizures (5.7%) (*p* = 0.031). Furthermore, patients with seizures had significantly higher rates of frontal shunt placement (63.6%) compared to those without seizures (20.7%) (*p* = 0.023). Conversely, the rate of occipital shunt placement was lower in patients with seizures (27.3%) compared to those without seizures (64.6%), with a *p* value of 0.023. Notably, patients with seizures did not significantly differ from those without seizures in terms of most neuroimaging findings, as evidenced by *p* values above 0.05. However, there was a significantly higher prevalence of corpus callosum dysgenesis (72.7%) in patients with seizures compared to those without seizures (29.3%) (*p* = 0.004).

Table 2 shows the relationship between shunt location and the history of shunt infection, as well as the number of shunt revisions. Patients with frontal shunt placement had a significantly higher incidence of shunt infection (50%) compared to those with parietal or occipital shunts (*p* ≤ 0.001). Moreover, these patients had a higher median number of shunt revisions (2) (*p* ≤ 0.001). In contrast, patients with parietal shunts had a relatively lower incidence of shunt infection (9.4%) (*p* = 0.024). The median number of shunt revisions in patients with parietal shunt (1) did not show a statistically significant difference (*p* = 0.339). Furthermore, patients with occipital shunts had a significantly higher incidence of shunt infection (40.6%) compared to those with parietal shunts (*p* ≤ 0.001), while their median number of shunt revisions (0) was statistically significant (*p* = 0.003). Both frontal and occipital shunt patients had significant effect sizes of more than 0.3 when calculated by the rank–biserial correlation in relation to the number of shunt revisions. Moreover, patients with occipital shunt had a Cramér's V of 0.305, indicating a significant effect size.

The characteristics of seizures observed in patients with MMC (*n* = 11) are presented in Table 3.

Among the patients with available data (*n* = 9), the majority of seizures were generalized (77.8%), while a smaller proportion were focal (22.2%). The median number of seizures among patients with available data (*n* = 9) was 1, with an interquartile range of 1. The median age of onset of the first seizure among patients with available data (*n* = 9) was 1.42 years, with an interquartile range of 1.73. Several anti-epileptic medications were administered to patients with available data (*n* = 9). Valproic acid (33.3%) and phenobarbital (55.6%) were the most commonly prescribed medications, while smaller proportions of patients received phenytoin (11.1%), levetiracetam (22.2%), and carbamazepine (11.1%).

### Predicting Seizures using ML

**Figure 2.**
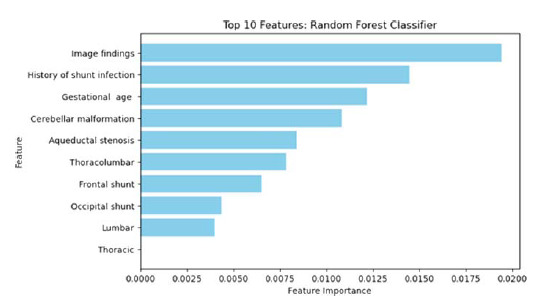
Permutation feature importance method determining the most contributing factors in the RFC model.

All metrics that evaluated the ML algorithms are presented in Table 4. The RFC model was performed on the testing set, which had a mean bootstrap estimate of 0.89 with a 95% CI of [0.81–0.97], a 10-fold cross-validation of 0.893, an accuracy of 0.87, and an AUC of 0.856. In contrast, the SVM model predicted on the testing set had a mean bootstrap estimate of 0.84 with a 95% CI of [0.72–0.95], a 10-fold cross-validation of 0.866, an accuracy of 0.85, and an AUC of 0.749. The LR model had a mean bootstrap estimate of 0.86 with a 95% CI of [0.75–0.95], a 10-fold cross-validation of 0.856, an accuracy of 0.87, and an AUC of 0.800. The ROC curves of all MLMs are shown in Figure 1. The most contributing features in predicting seizures in the RFC model are shown in Figure 2, with image findings identified as the highest contributing factor, followed by the history of shunt infection and gestational age.

## Discussion

Seizures are commonly observed in individuals born with MMC. In cases involving HC, the occurrence of seizures has been reported to vary between 14.7% and 29%. Conversely, children with MMC but without shunts experience a significantly lower incidence of seizures, approximately ranging from 2% to 8%.^11^ Notably, certain studies have demonstrated a correlation between the occurrence of seizures in the MMC population and unfavorable neurodevelopmental outcomes, coupled with more pronounced brain malformations.^12,13,15–17^ A significant proportion, ranging from 6% to 59%, of children requiring ventricular shunts face an elevated risk of developing seizures compared to the general population, as shown by various investigations.^12,18–32^ The increased susceptibility to seizures in this context may be attributed to several contributing factors, including HC, treatment modalities, and any associated complications. Findings from multiple studies suggest that factors such as shunt insertion, the frequency of shunt revisions, a history of shunt infection, and possibly the location of the shunt can collectively increase the risk of seizures.^22,33–35^

This study revealed a significant association between gestational age and the risk of seizures in patients with MMC. Notably, among the 10 patients who experienced seizures, 60% were born preterm, while only 20.5% of the 78 patients without seizures were preterm. This finding is consistent with previous research and establishes a statistically significant association. Previous studies have consistently highlighted that preterm infants are at an increased risk of seizures, with rates as high as 22.2%, compared to a significantly lower incidence of 0.5% in full-term infants.^36,37^

The significance of MMC location in our study was evident in the sacral region in relation to seizures. This finding contradicts two previous studies, which reported a significant increase in the risk of seizures associated with lumbar MMC. However, these studies did not provide an explanation for this observed relationship.^13,38^

HC is a common complication associated with MMC, affecting approximately 65–85% of individuals.^39–41^ Our findings are consistent with this trend, as 65 out of 92 patients (70.7%) without seizures developed HC. Conversely, all 11 patients (100%) with seizures presented with HC. The location of MMC plays a significant role in determining the incidence of HC, as highlighted by Laurence and Lorber. According to their findings, the risk of HC is highest when MMC is located in the lumbar region (80%), followed by the thoracolumbar region (70%), the sacral region (50%), and the thoracic region that exhibits the lowest risk (43%).^40,41^

Chadduck and Adametz proposed various etiologies that may increase the risk of seizures in patients requiring shunt placement. One such factor is the insertion of the tube into the ventricular system, which may induce cortical injury. Shunt-related complications, including infection and malfunction, are identified as additional risk factors for the development of seizures. Notably, the underlying indication for the need for shunt placement emerges as an important etiological factor, potentially resulting in the most significant influence on seizure susceptibility.^10^ In a study conducted by Bourgeois et al. involving patients with HC, including those with MMC, it was found that 28.6% of patients who experienced seizures had their initial seizure before shunt insertion. In contrast, seizures were reported in the remaining 71.4% of cases following the insertion of the shunt, indicating a significant impact of the shunting procedure on the onset of epilepsy. Nevertheless, other studies suggest that there is no significant difference in the incidence of epilepsy between MMC patients with and without HC. This finding emphasizes the potential role of underlying pathological conditions in influencing these outcomes.^12,17,33^

The increase in seizure rates among patients undergoing recurrent shunt revisions can be attributed to two primary factors. Firstly, each shunt insertion has the potential to cause cortical injury. The cumulative effect of multiple shunt procedures may contribute to an elevated risk of developing seizures.^10^ This association emphasizes the importance of considering the procedural impact on cortical structures and their risk for neurological outcomes. Secondly, the increase in intracranial pressure, particularly when it remains uncontrolled, is considered another significant contributor to the increased incidence of seizures observed in this study. Elevated intracranial pressure, a potential consequence of recurrent shunt revisions, is believed to induce damage, further emphasizing the balance required in managing intracranial pressure. Both mechanisms involving direct cortical impact and alterations in intracranial pressure illustrate the relationship between shunt revisions and seizure susceptibility.^10^

As the pioneering study in developing an ML model to predict seizures in MMC patients, our research holds importance in advancing medical care for MMC patients. This predictive tool has the potential to transform patient management by facilitating early identification of individuals who are at an increased risk of seizures. By using advanced algorithms to analyze patient data, the model could offer valuable insights for physicians, enabling targeted interventions for improved patient care. Early detection and personalized interventions may lead to a reduction in both the frequency and severity of seizures, ultimately enhancing outcomes for individuals with MMC.

The limitations of our study include a relatively small sample size, which highlights the need for future investigations involving larger and more diverse cohorts to strengthen the statistical significance of the observed variables and to further clarify the relationship between seizures and MMC. Additionally, a larger sample size is crucial for refining the predictive ability of our machine learning model, thereby improving its robustness and generalizability. The external validation of our pioneering ML model is essential, as it represents a novel approach in this field. The development of additional models by different research groups would contribute to broader validation. Moreover, since our research is based on a single-center study, our findings may have inherent biases, necessitating caution in generalizing the results. Therefore, conducting multi-center studies would provide a more comprehensive understanding of seizures in patients with MMC.

## Conclusion

In this study, ML techniques effectively predicted the risk of seizures in MMC patients, achieving accuracy rates between 86% and 92% and AUC values between 0.764 and 0.865. Key predictors included image findings, a history of shunt infection, and gestational age. Descriptive analysis revealed multiple variables that influence the risk of seizures, including gestational age, sacral MMC, HC, shunt placement, number of shunt revisions, a history of shunt infection, and corpus callosum dysgenesis. A significant relationship was observed between shunt location and the history of shunt infection. Additionally, a relationship was observed between shunt location and the number of shunt revisions, with frontal VP shunts associated with a higher median number of shunt revisions and occipital shunts linked to a lower median number of revisions. Effect size measurements emphasized strong associations and significant effect sizes for multiple variables.

### Authors' contribution

All authors made a significant contribution to this work, whether in the conception, study design, execution, data acquisition, analysis, and interpretation, or in all these areas. They participated in drafting, revising, or critically reviewing the manuscript; gave final approval for the version to be published; consented to the journal to which the article has been submitted; and accepted responsibility for all aspects of the work.

### Ethics approval

This study was approved by the ethics committee of Jordan University of Science and Technology.

### Competing interests

The authors declare that they have no competing interests.

## Figures and Tables

**Table 1.** Demsographic and clinical characteristic analysis in MMC patients: patients without seizures versus patients with seizures.

Patient characteristics, *n* (%) (total *n* with available data)	All patients (*n*=103)	No seizure (*n*=92)	Seizure (*n*=11)	*p*	Cramér’s V/rank-biserial correlation

Demographics (*n*=103)	(*n*=103)	(*n*=92)	(*n*=11)		

Gender				0.107	0.159

Female	61 (59.2)	52 (56.5)	9 (81.8)		

Male	42 (40.8)	40 (43.5)	2 (18.2)		

Age, median (IQR)	7 (4.0)	7.00 (4.0)	8 (1.5)	0.178	0.249

Perinatal history (*n*=88)	(*n*=88)	(*n*=78)	(*n*=10)		

Gestational age, preterm	22 (25.0)	16 (20.5)	6 (60)	0.018^*^	0.289

Mode of delivery (cesarean)	66 (75.0)	57 (73.1)	9 (90)	0.422	0.124

MMC level (*n*=98)	(*n*=98)	(*n*=87)	(*n*=11)		

Thoracic	3 (3.1)	2 (2.3)	1 (9.1)	0.331	0.124

Thoracolumbar	16 (16.3)	13 (14.9)	3 (27.3)	0.414	0.105

Lumbar	32 (32.7)	29 (33.3)	3 (27.3)	0.671	0.041

Lumbosacral	32 (32.7)	31 (35.6)	1 (9.1)	0.143	0.179

Sacral	8 (8.2)	5 (5.7)	3 (27.3)	0.031^*^	0.248

Hydrocephalus (*n*=103)	76 (73.8)	65 (70.7)	11 (100)	0.036^*^	0.206

Shunt placement (*n*=103)	76 (73.8)	65 (70.7)	11 (100)	0.036^*^	0.206

Frontal shunt	26 (33.8)	19 (29.2)	7 (63.6)	0.023^*^	0.258

Parietal shunt	9 (11.7)	8 (12.3)	1 (9.9)	0.389	0.033

Occipital shunt	45 (58.4)	42 (64.6)	3 (27.3)	0.023^*^	0.258

Shunt history (*n*=103)	(*n*=103)	(*n*=92)	(*n*=11)		

Number of shunt revisions, median (IQR)	0.00 (1.00)	0.00 (1.00)	2.00 (1.50)	< 0.001^*^	0.573^*^

History of shunt infection	32 (31.1)	24 (26.1)	8 (72.7)	0.002^*^	0.311^*^

Neuroimaging findings (*n*=103)	73 (70.9)	64 (69.6)	9 (81.8)	0.398	0.083

Suprasellar arachnoid cyst	1 (1.0)	1 (1.1)	0 (0)	0.728	0.034

Absent septum pellucidum	15 (14.6)	13 (14.1)	2 (18.2)	0.791	0.035

Corpus callosum dysgenesis	35 (34.0)	27 (29.3)	8 (72.7)	0.004^*^	0.283

Cerebellar malformation	64 (62.1)	59 (64.1)	5 (45.5)	0.227	0.119

Falx dysgenesis	4 (3.9)	3 (3.3)	1 (9.1)	0.344	0.093

Aqueduct stenosis	2 (1.9)	2 (2.2)	0 (0)	0.621	0.049

Intracranial cyst	1 (1.0)	1 (1.1)	0 (0)	0.728	0.034

Subdural collection	1 (1.0)	1 (1.1)	0 (0)	0.728	0.034

Enlarged subarachnoid space	2 (1.9)	2 (2.2)	0 (0)	0.621	0.049

Heterotopia	2 (1.9)	2 (2.2)	0 (0)	0.621	0.049


Note: ^*^Statistical significance was determined with a *p* value ≤ 0.05. Cramér's V or rank–biserial correlation values ≥ 0.3 indicated a statistically significant effect size. Descriptive measures included a median with the interquartile range since the normality assumption was violated according to the Shapiro–Wilk test. For analysis of *p* values, the chi-square test was used for categorical variables, while the Mann–Whitney U test was used for non-normally distributed numerical variables. Effect sizes were calculated using the rank–biserial correlation for numerical variables and Cramér's V for categorical variables.

IQR: interquartile range (Q3–Q1), MMC: myelomeningocele.

**Table 2.** Relationship of shunt location with the history of shunt infection and the number of shunt revisions.

Shunt location (*n*=76)	History of shunt infection, *n* (%) (*n*=32)	*p*	Cramér’s V	Number of shunt revisions, median (IQR)	*p*	Rank-biserial correlation

Frontal (*n*=26)	16 (50)	< 0.001^*^	0.289	2.00 (2.00)	< 0.001^*^	0.533^*^

Parietal (*n*=9)	3 (9.4)	0.024^*^	0.061	1.00 (4.00)	0.339	0.190

Occipital (*n*=45)	13 (40.6)	< 0.001^*^	0.305^*^	0 (1.00)	0.003^*^	0.379^*^


Note: ^*^Statistical significance was determined with a *p* value ≤ 0.05. Cramér's V or rank–biserial correlation values ≥  0.3 indicated a statistically significant effect size. Descriptive measures included a median with the interquartile range since the normality assumption was violated according to the Shapiro–Wilk test. For analysis of *p* values, the chi-square test was used for categorical variables, while the Mann–Whitney U test was used for non-normally distributed numerical variables. Effect sizes were calculated using the rank–biserial correlation for numerical variables and Cramér's V for categorical variables.

IQR: interquartile range (Q3–Q1).

**Table 3.** Characteristics of seizures.

Characteristics of seizures (total *n* with available data)	Patients with seizures (*n* =11)

Type of seizure (*n*=9)	

Focal, *n* (%)	2 (22.2)

Generalized, *n* (%)	7 (77.8)

Number of seizures, median (IQR) (*n*=9)	1.00 (1.00)

Age of onset of the first seizure, median (IQR) (*n*=9)	1.42 (1.73)

Medications (*n*=9)	

Valproic acid, *n* (%)	3 (33.3)

Phenobarbital, *n* (%)	5 (55.6)

Phenytoin, *n* (%)	1 (11.1)

Levetiracetam, *n* (%)	2 (22.2)

Carbamazepine, *n* (%)	1 (11.1)


Note: Descriptive measures included a median with the interquartile range since the normality assumption was violated according to the Shapiro–Wilk test.

IQR: interquartile range (Q3–Q1).

**Table 4.** Performance of machine learning algorithms.

ML algorithm	10-fold cross-validation	Accuracy	Mean bootstrap estimate [95% CI]	AUC

RFC	0.893	0.87	0.89 [0.81-0.97]	0.856

SVM	0.866	0.85	0.84 [0.72-0.95]	0.749

LR	0.856	0.87	0.86 [0.75-0.95]	0.800


ML: machine learning, CI: confidence Interval, AUC: area under the curve, RFC: random forest classifier, SVM: support vector machine, LR: logistic regression.
